# Real‐time surveillance systems: Applicability for the control of influenza in acute care

**DOI:** 10.1111/irv.12720

**Published:** 2020-03-02

**Authors:** Víctor Quirós‐González, Paz Rodríguez‐Pérez, Ana Mª Haro‐Pérez, Mª Mar Jiménez‐Rodríguez, José Ángel Maderuelo‐Fernández, José Mª Eiros

**Affiliations:** ^1^ Department of Preventive Medicine Institute of Biomedical Research of Salamanca (IBSAL) University Hospital of Salamanca Salamanca Spain; ^2^ Primary Health Care Research Unit of Salamanca APISAL Institute of Biomedical Research of Salamanca (IBSAL) Health Service of Castilla y León (SACYL) Salamanca Spain; ^3^ Department of Clinical Microbiology University Hospital Rio Hortega Valladolid Spain

**Keywords:** cross‐infection, influenza, microbiology, preventive medicine, vaccination

## Abstract

**Background:**

The high morbidity and mortality caused by influenza viruses translate into a great impact on specialized health care. Apart from the annual vaccination, the relevance of other measures to prevent and control this infection is unknown. The objective of our research was to determine the importance of a real‐time surveillance system to establish early extended transmission precautions.

**Methods:**

Quasi‐experimental before‐and‐after study comparing the influenza cases detected in hospitalized adults during the 2016/2017 season (264 patients) with those detected after the implementation of a real‐time surveillance system in the 2017/2018 season (519 patients). The improvements included early microbiological diagnosis, immediate communication of results, constant updating of patient information, coordination among professionals, periodic surveillance of the adequacy of preventive measures, and greater control of roommates. The effectiveness of the intervention was determined from the nosocomial infection rate in each season.

**Results:**

After the real‐time surveillance system for influenza was implemented, patients with early microbiological diagnosis and immediate isolation increased significantly (13.7% vs 68.2%; *P* < .001). In addition, nosocomial infections decreased from 17% to 9.2% (*P* = .001) and overall hospital stay was significantly reduced. Assuming that the entire effect was due to the intervention, the absolute risk reduction was 7.8% and number needed to treat was 12.8.

**Conclusion:**

The results in our study reveal the impact of nosocomial transmission of influenza virus in a tertiary hospital and highlight the need to supplement traditional strategies with novel methodologies such as modern surveillance systems based on early diagnosis, close case monitoring, and coordination among professionals.

## 1 INTRODUCTION

Influenza viruses have a great importance in public health due to the high morbidity they cause and the associated mortality, both directly and by aggravation of other diseases.^1^ Although the entire population is susceptible to getting an influenza virus infection, incidence, morbidity, and mortality are higher in certain risk groups, such as people over 60 years old, children under 2 years old, patients with chronic diseases, immunosuppressed people, and pregnant women.^2^, ^3^

Influenza activity in Spain during the 2016/2017 (S1617) and 2017/2018 (S1718) seasons was considered moderate. S1617 was associated with the transmission of almost absolutely influenza A virus (H3N2),^4^, ^5^ while S1718 was characterized by a mixed circulation, with a predominance of influenza B.^6^ The high incidence of this pathology and its complications determined a notable increase in the demand for care.^7^ Previous research places the costs associated with a flu season above 1 billion euros. One‐quarter of the total correspond to direct health expenses, while the remaining three‐quarters are associated with labor costs.^8^, ^9^ The numerous nosocomial outbreaks described in hospital rooms, adult and neonatal intensive care units, transplant centers, and other chronic care centers increase the frequency of influenza cases related to health care to 15%.^10^

Being the most frequent immunopreventable disease in developed countries, the main strategy for the prevention and control of influenza is vaccination, in a common effort of public health authorities, health professionals, and patients.^1^ Sufficient evidence supports the vaccination of health professionals as one of the most important measures in the reduction of nosocomial transmission of influenza virus.^11^, ^12^, ^13^ However, as reflected in various recommendations,^14^, ^15^ health centers must develop other actions to minimize possible complications in the infected patient and avoid cross‐transmission to susceptible people. These measures include the encouragement of compliance with standard precautions (with special attention to hand hygiene), the early implementation of extended precautions for transmission by droplets, environmental infection control and engineering controls, training and education of healthcare personnel, and administration of antiviral treatment and chemoprophylaxis of patients and healthcare personnel when appropriate.^16^

One of the specific recommendations of the Centers for Disease Control and Prevention (CDC)^15^ is the monitoring of influenza activity, understood as communication and collaboration with local and state health authorities, but also the control of outbreaks that may occur in health centers. At this point, experience in surveillance systems is greater for multidrug‐resistant microorganisms than for acute respiratory virus infections.^17^, ^18^ Despite the obvious epidemiological differences between these groups, we believe that this approach could also be applicable to influenza. In this sense, it has been proposed not to limit the approach to pharmacological measures, but to include the close monitoring of cases with updated information, verifying the quality of the interventions, taking special care isolating patients, and applying a multidisciplinary approach and constant coordination.^19^

Although each measure (patient vaccination, professional vaccination, hand hygiene, patient isolation, early antiviral treatment, and use of masks) would limit the transmission of the virus on its own,^20^ the use of combined strategies would reduce nosocomial transmission by half.^21^ These estimates obtained by predictive mathematical models would have to be corroborated by evaluating multicomponent interventions in real environments. As far as we know, the effectiveness of an intervention that integrates the aforementioned strategies into a single surveillance system encompassing and coordinating multiple professionals from different specialties has not been evaluated.

Therefore, the objective of this study was to determine the impact of the implementation of a real‐time surveillance system, similar to that used in the monitoring of multidrug‐resistant microorganisms, on nosocomial transmission of influenza viruses as evaluated by the nosocomial infection rate.

## 2 METHODS

### 2.1 Study design

Quasi‐experimental before‐and‐after study, in which we compared the cases of influenza detected in adults hospitalized during S1617 and those identified after the implementation of a real‐time surveillance system in S1718.

### 2.2 Setting

The study took place in a tertiary hospital with 907 beds in the province of Salamanca, Castilla y León (Spain). The total number of discharges during 2016 was 31 366, with an average stay of 7.12 days and 157 758 emergencies, of which 12.63% required admission. During 2017, the total number of discharges was 33 336, with an overall average stay of 6.85 days and a total of 155 288 emergencies, of which 12.58% required admission.

In addition to special epidemiological situations, vaccination against influenza is recommended in the population over the age of 60 in Spain.^22^ Castilla y León presented one of the highest influenza vaccination coverages in Spain in this group (over 60% in both seasons).^23^ The vaccination rate against influenza virus among health professionals in our center was 29% in S1617 and 28% in S1718.

The specific epidemiological surveillance system of the center, used in S1617 and previous campaigns, consisted in evaluating patients who come to the emergency department with respiratory symptoms, identifying those potentially infected and putting on a surgical mask. When the patient required hospitalization, extended precautions for droplet transmission were used, isolating the patient in an individual room. If these rooms were not available, the cohort was isolated in double rooms with 2 patients with the same type of virus, guaranteeing more than 1 meter of distance between beds. Additionally, the main measures for preventing the transmission of pathogenic microorganisms were intensified: extreme hand hygiene of people in contact with the patient and their environment, limiting the number of workers, visitors and relatives exposed to these patients, and reducing, as far as possible, the movement of patients within the hospital. The treatment of waste and the cleaning and disinfection of the environment were carried out following the usual protocol in our center.

### 2.3 Intervention: improvements in the surveillance system

**Figure 1:**
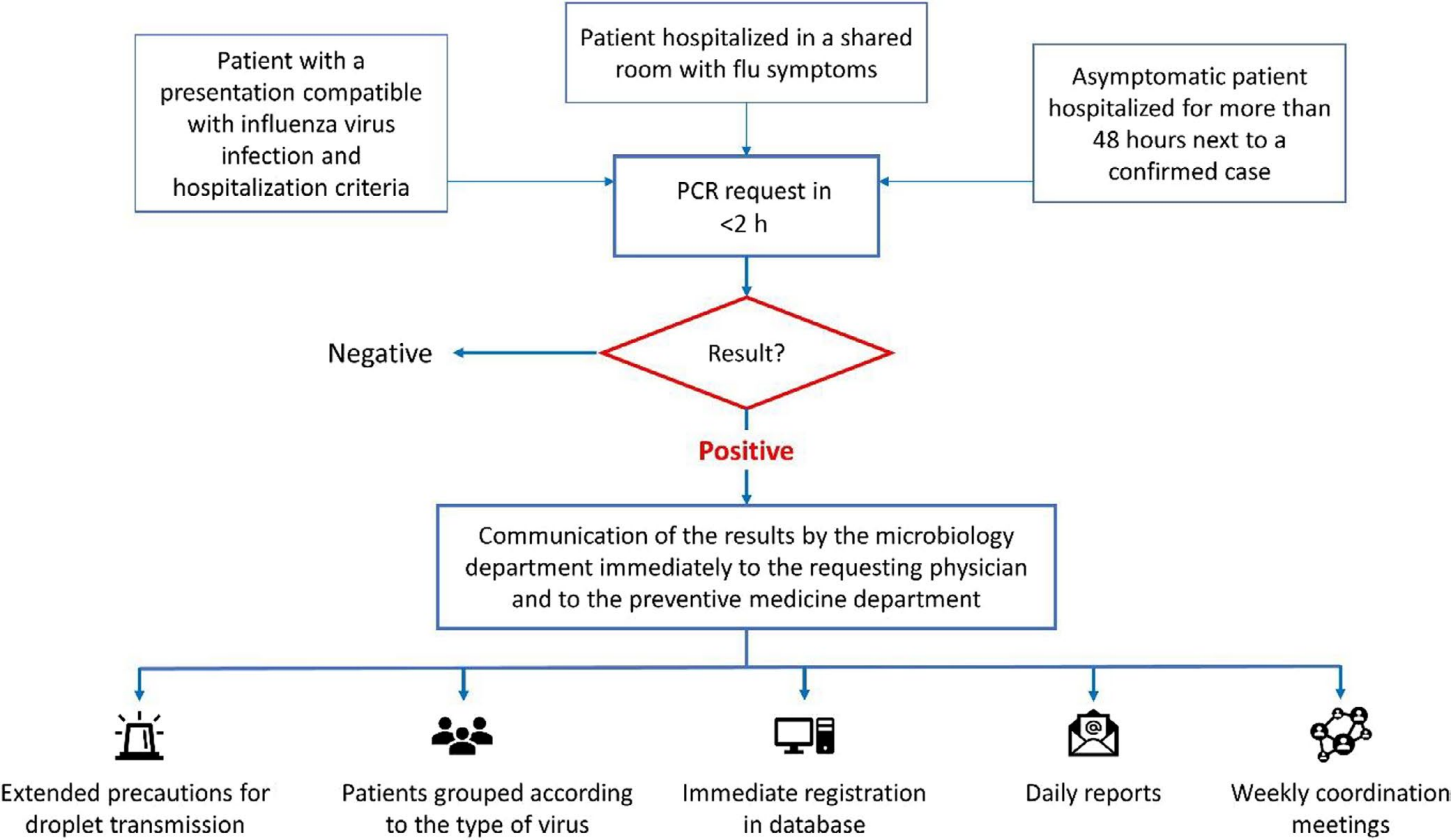
Action algorithm of the influenza control and surveillance system

Before the start of S1718, the action protocol against influenza in adults was modified, with prospective collection of information and immediate decision‐making. The new real‐time surveillance system (Figure 1) maintained the prevention and control measures of previous seasons, and incorporated the following developments: (a) **request for microbiological tests**. Performing PCR in less than 2 hours after identification of patients with a presentation compatible with influenza virus infection and hospitalization criteria. In S1617, "early request" was mentioned without a definite time criterion; (b) **communication of the results** by the microbiology department immediately to the requesting physician by telephone and to the doctors responsible for the control of nosocomial infection through email; (c) **registration and collection of information**. The preventive medicine department recorded the reported cases, in real time, in a specific computer system. In addition, the patient's location was updated twice a day and possible patient transfers were recorded for testing; (d) **coordination among professionals**. First, daily reports were sent to the hospital management team, admissions and all departments involved, at 9:00 am and 3:00 pm, so that all groups had the same updated information regarding admissions and discharges (Appendix S1). These reports listed hospitalized patients in the period of transmissibility of the infection, their location, and the type of transmission (nosocomial/community). In addition, every Friday, weekly coordination meetings were held between the management team and the professionals of the departments of internal medicine, infectious diseases, microbiology, and preventive medicine, in which the epidemiological situation of the flu in the center was discussed and decisions were made on measures to be taken from the beginning of the following week (eg, blocking of beds in anticipation of a significant increase in the number of admissions, redistribution of professionals, etc); (e) **follow‐up of hospitalization** with periodic visits by professionals of healthcare‐associated infection control to verify compliance with established precautions and computer alerts after 5 days of antiviral treatment in order to avoid unnecessary prolongations of isolation; and (f) **roommate control.** At occurrence of a case of nosocomial flu, the roommate was tested. If a patient had been hospitalized for more than 48 hours next to a confirmed case, a PCR was requested. In case of a positive result, given the same type of virus, the precautions for droplet transmission were put in place for both patients. If the result was negative, the patient was changed to a different room immediately.

### 2.4 Study population

All adults admitted with confirmed influenza were included (264 cases during S1617 and 519 cases during S1718; total = 783 patients), with a positive test for viral RNA in respiratory samples, identified through the center's epidemiological surveillance program of influenza cases. We excluded positive PCRs of pediatric patients (22 in S1617 and 15 in S1718).

### 2.5 Definition of confirmed influenza case

We defined case as a patient with influenza syndrome (at least one of the following four general symptoms: fever or high‐grade fever, malaise, headache, myalgia; and at least one of the three respiratory symptoms: cough, sore throat, dyspnea; and absence of differential diagnosis), confirmed through the PCR detection of viral RNA in respiratory samples processed in real time in the microbiology laboratory. Influenza of nosocomial origin was defined in the cases that developed influenza syndrome after 72 hours after admission or who re‐entered with influenza symptoms within 72 hours after discharge.

### 2.6 Study variables

The variables included in our study were sex, age, flu season, vaccination for S1617, vaccination for S1718, time elapsed since vaccination until the positive PCR result, type of infection (nosocomial/community), admission in intensive care, situation at discharge (improvement/success), and days of stay from the microbiological diagnosis of the infection to discharge.

### 2.7 Statistical analysis

The data were analyzed based on the computerized record included in our center's global strategy for surveillance and control of influenza. After checking the normality of the distribution of the values in the sample through the Shapiro‐Wilk test, we conducted the association study in two phases. First, we examined the relationship between variables within the same season. We then examined whether the values changed between the two seasons, before and after the implementation of the real‐time surveillance system. The association between categorical variables was studied with the chi‐square test and Fisher's exact test, while for quantitative variables, we used Student's *t* test. Finally, the effect of the intervention was determined calculating the absolute risk reduction (ARR) and the number of patients needed to treat (NNT). *P*‐values below .05 were considered statistically significant. We used IBM SPSS Statistics Version 23.

## 3 RESULTS

Overall, 783 adult patients required hospitalization in both seasons due to influenza virus infection, of which 485 cases (61.9%) corresponded to influenza A and 298 (38.1%) to influenza B. The attributable mortality was 10.3%. According to the case grouping strategy, 58.3% of the patients were admitted by the internal medicine department and 5.4% needed to be admitted to the ICU. Based on the established criteria, 93 cases (11.9%) were considered nosocomial and 690 (88.1%) community infections. Nosocomial cases differed from community cases in the number of ICU admissions (nosocomial: 14.0%; community: 4.2%, *P* < .001), mortality (nosocomial: 17.2%; community: 9.4%, *P* = .03), hospital stay after microbiological diagnosis (nosocomial: 11 ± 16 days; community: 7 ± 6 days, *P* < .001), and vaccination rate (nosocomial: 34.4%; community: 53.0%, *P* = .001). The overall vaccination coverage was 50.8%, with 55.7% of adults vaccinated among those who had specific recommendations due to age. The specific characteristics of adults hospitalized in both seasons are shown in Table 1.

**Table 1:** Characteristics of admitted patients with PCR‐confirmed influenza in S1617 and S1718

	Season 2016/2017					Season 2017/2018				
	Nosocomial		Community		*P*‐value	Nosocomial		Community		*P*‐value
	n (45)	% (17.0)	n (219)	% (83.0)		n (48)	% (9.2)	n (471)	% (90.8)	
Sex										
Male	30	66.7	122	55.7	.18	30	62.5	230	48.8	.07
Female	15	33.3	97	44.3		18	37.5	241	51.2	
Age group										
≥60 y	37	82.2	199	90.9	.11	37	77.1	407	86.4	.08
<60 y	8	17.8	20	9.1		11	22.9	64	13.6	
Age										
Mean (SD)	76	(±15)	79	(±15)	.15	72	(±14)	77	(±16)	**.03**
Vaccination										
Yes	15	33.3	115	52.5	**.02**	17	35.4	251	53.3	**.02**
No	30	66.7	104	47.5		31	64.6	220	46.7	
Admission to ICU										
Yes	7	15.6	10	4.6	**.01**	6	12.5	19	4.0	**.02**
No	38	84.4	209	95.4		42	87.5	452	96.0	
Status on discharge										
Improvement	36	80.0	201	91.8	**.03**	41	85.4	424	90.0	.22
Death	9	20.0	18	8.2		7	14.6	47	10.0	
Total days of stay										
Mean (SD)	21	(±24)	8	(±8)	**<.001**	15	(±10)	7	(±4)	**<.001**
Days of stay after PCR confirmation										
Mean (SD)	12	(±21)	7	(±8)	**<.001**	11	(±10)	7	(±4)	**<.001**

Note

In bold, statistically significant values (*P* < .05).

Abbreviations: PCR, polymerase chain reaction; SD, standard deviation.

Between November 1, 2016, and March 31, 2017 (S1617), 63 492 patients were treated, of whom 51 380 were adults and 12 112 pediatric patients. The total number of PCR tests for influenza viruses that were performed in the hospital was 1359. In the same period of the following season (S1718), 65 959 patients (52 463 adults and 13 496 children) were attended to by the emergency department and 3054 PCR were requested. The proportion of positive PCR differed between seasons (S1617: 21%; S1718: 17.5%, *P* = .006). As Table 2 shows, the percentage of community cases with microbiological diagnosis from the emergency department increased significantly in the intervention season, with the consequent early isolation in a single room. The waiting time from arrival to hospitalization for these patients was also longer in S1718 (5.3 vs 6.4 hours; *P* = .03).

**Table 2:** Comparison of patients admitted with viral influenza during two seasons

	Total patients					Nosocomial cases				
	S1617		S1718		*P*‐value	S1617		S1718		*P*‐value
	n (264)	% (31.3)	n (519)	% (68.7)		n (45)	% (17.0)	n (48)	% (9.2)	
Type of infection										
Nosocomial	45	17.0	48	9.2	**.001**	—				
Community	219	83.0	471	90.8						
Location of patient at microbiological diagnosis										
Emergency room	30	13.7	321	68.2	**<.001**	—				
Hospitalized	189	86.3	150	31.8						
Waiting hours from arrival to hospitalization										
Mean (SD)	5.3	(±3.5)	6.4	(±4.2)	**.03**	—				
Virus strain										
A	264	100	221	42.6	**<.001**	45	100	16	33.3	**<.001**
B	—	—	298	57.4		—	—	32	66.7	
Sex										
Male	152	57.6	260	50.1	**.04**	30	66.7	30	62.5	.42
Female	112	42.4	259	49.9		15	33.3	18	37.5	
Age										
Mean (SD)	79	(±15)	77	(±16)	**.04**	76	(±15)	72	(±14)	.16
Age group										
≥60 y	236	89.4	444	85.6	.13	37	82.2	37	77.1	.36
<60 y	28	10.6	75	14.4		8	17.8	11	22.9	
Place of stay										
Admitted	67	25.4	107	20.6	.15	10	22.2	6	12.5	.17
Home	197	74.6	412	79.4		35	77.8	42	87.5	
Vaccination										
Yes	130	49.2	268	51.7	.53	14	31.1	17	35.4	.41
No	134	50.8	251	48.3		31	68.9	31	64.6	
Vaccination for age recommendation										
Yes	127	53.8	252	56.8	.46	13	36.1	16	43.2	.35
No	109	46.2	192	43.2		23	63.9	21	56.8	
Admission to ICU										
Yes	17	6.4	25	4.8	.34	7	15.6	6	12.5	.45
No	247	93.6	494	95.2		38	84.4	42	87.5	
Status at discharge										
Improvement	237	89.8	465	89.6	.94	36	80.0	41	85.4	.34
Death	27	10.2	54	10.4		9	20.0	7	14.6	
Total days of stay										
Mean (SD)	10	(±13)	8	(±6)	**<.001**	21	(±24)	15	(±10)	.13
Days of stay after PCR confirmation										
Mean (SD)	7	(±11)	7	(±5)	.61	12	(±21)	11	(±10)	.77

Note

In bold, statistically significant values (*P* < .05).

Abbreviations: PCR, polymerase chain reaction; S1617, season 2016/2017; S1718, season 2017/2018; SD, standard deviation.

**Figure 2:**
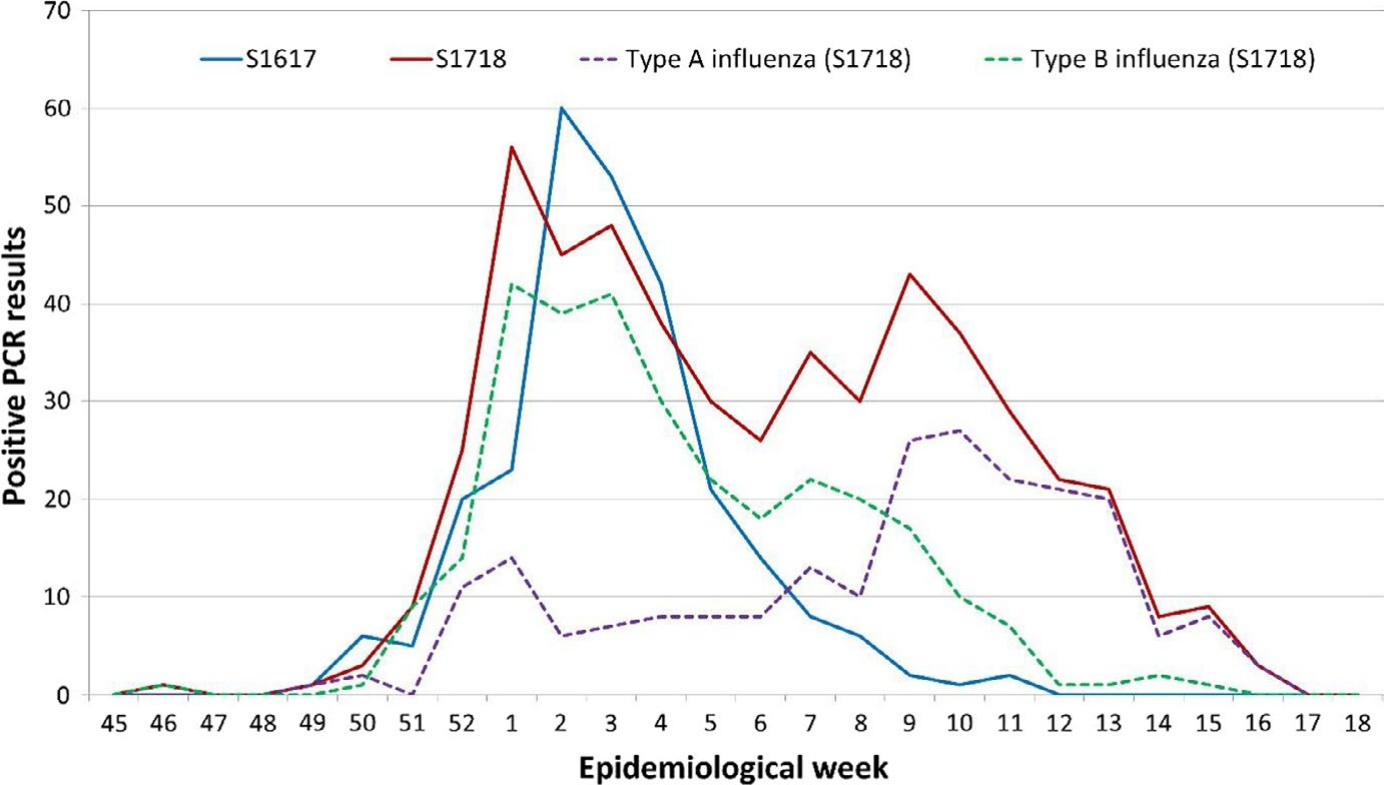
Detection of positive cases of influenza virus by epidemiological week

The epidemic curve during the S1617 campaign (Figure 2) showed the highest incidence during the second epidemiological week with 60 notifications between January 8 and 14, all detections corresponding to virus A. During the S1718 campaign, the epidemic curve reached the maximum level of weekly notifications (56) during the 1st epidemiological week of 2018, from January 1 to 7. Depending on the type of influenza virus, the peak incidence of influenza B occurred in the 1st epidemiological week accounting for 42 notifications, while for influenza A, it was in the tenth epidemiological week with 27 notifications between March 4 and 10, 2018. We only found differences between the types of virus in the time since vaccination until a positive PCR result (Table 3).

**Table 3:** Characteristics of admitted patients by viral strain during S1718

	Influenza A		Influenza B		*P*‐value
	n (221)	% (42.6)	n (298)	% (57.4)	
Sex					
Male	118	53.4	142	47.7	.2
Female	103	46.6	156	52.3	
Age group					
≥60 years	194	87.8	250	83.9	.2
<60 years	27	12.2	48	16.1	
Age					
Mean (SD)	78	(±15)	76	(±17)	.2
Type of infection					
Nosocomial	16	7.2	32	10.7	.1
Community	205	92.8	266	89.3	
Vaccination					
Yes	115	52.0	153	51.3	.9
No	106	48.0	145	48.7	
Days since vaccination until a positive PCR result					
Mean (SD)	111	(±29)	79	(±28)	**<.001**
Admission to ICU					
Yes	11	5.0	14	4.7	.9
No	210	95.0	284	95.3	
Status at discharge					
Improvement	197	89.1	268	89.9	.8
Death	24	10.9	30	10.1	
Total days of stay					
Mean (SD)	8	(±6)	8	(±6)	.9
Days of stay after PCR confirmation					
Mean (SD)	7	(±5)	7	(±5)	.6

Note

In bold, statistically significant values (*P* < .05).

Abbreviations: PCR, polymerase chain reaction; SD, standard deviation.

After the implementation of the real‐time influenza surveillance system, nosocomial virus transmission was reduced significantly by 7.8%, from 17.0% in S1617 to 9.2% in S1718. Likewise, the duration of hospital stays decreased, with no differences in the vaccination rate between seasons. These results, together with the impact on nosocomial cases, are shown in Table 2. Assuming that the entire effect was due to the intervention, we obtained an ARR of 7.8%, meaning that for every 100 patients in whom the real‐time surveillance system was implemented, almost 8 cases of nosocomial influenza were averted. The NNT was 12.8, meaning we would have to implement the system in 13 patients to avoid 1 case of nosocomial transmission.

## 4 DISCUSSION

This study aimed to analyze the impact of implementing a real‐time surveillance system on influenza control. The results indicate an important reduction of 7.8% in nosocomial transmission of the virus after the intervention, as well as in the length of hospital stay of all patients.

Our results are coherent with global data on influenza activity in Spain. 98.6% of sentinel detections in S1617 were influenza A viruses, 100% in our center.^5^ During S1718, there was a predominance of influenza B (59.0% in national data compared to 57.4% of notifications of our microbiology department).^6^ The different temporal pattern of influenza B with respect to A could be due to various synergistic causes. The difference in days from vaccination to PCR diagnosis between the two types of virus and the known divergence in multiple seasons between the vaccine strain and the lineage of the circulating B strain^24^, ^25^ could cause an initial containment of influenza A that was not produced for the B strain.

Nosocomial transmission of influenza viruses has a great impact on specialized healthcare.^26^ The proportion in our center (11.9%) is consistent with figures published by hospitals of similar characteristics, varying from 4.3% to 17% in studies from Australia and the United Kingdom.^16^, ^27^, ^28^ In line with previous research, when comparing nosocomial and community cases, we noted a higher probability of admission to intensive care and an increased mortality in the former. Our experience placed that difference in mortality at 7.8%. In addition, contrary to results presented in other publications,^16^ we highlight the importance of vaccination in preventing this type of transmission: In our sample, 53.0% of cases of community‐acquired influenza had been vaccinated in the previous campaign, compared to 34.4% of nosocomial cases. Despite the great variations between seasons in the effectiveness of the vaccine (ranging from 10% to 90% protection^25^), its capacity is demonstrated by reducing the main complications associated with influenza, as well as the number of most serious cases.^29^ Similarly, vaccination of health professionals is one of the most effective resources in reducing the spread of seasonal flu in hospitals.^30^ Most European experiences place vaccination coverage in this group close to 30%, with a worrying decreasing trend.^13^ Given this scenario, it is important to increase efforts and design new strategies that allow professionals to raise awareness about the importance of vaccination to ensure patient safety.

However, and reiterating the commitment to this measure, we emphasize the specific impact of other fundamental actions, such as the development of surveillance systems, compliance with standard precautions and the establishment of extended precautions for droplet transmission.^15^ As part of our real‐time surveillance system, concrete strategies, such as the training of professionals, hand hygiene, or the use of masks, are protective factors for nosocomial transmission of influenza.^20^, ^31^ Similarly, the early microbiological diagnosis of the infection is effective both in the pediatric population^32^ and in the general population.^33^ In our case, more than two‐thirds of the patients hospitalized in S1718 with community flu already entered through the emergency department with a positive microbiological diagnosis, compared to 13.7% of the first season. This allowed the early isolation and presumably reduced the nosocomial transmission of the virus. It seems logical that the synergistic effect of these actions could boost the effect of each action on its own. Through predictive models, we know that combined strategies, including hand hygiene, surgical mask, patient isolation, antiviral treatment, and previous vaccination, could reduce the nosocomial transmission of the virus by half.^21^ Indeed, without our intervention affecting the vaccination of patients and professionals, the results place this decrease at 45.9%. The new system featured improvements such as closer surveillance of cases, coordination of professionals or extended surveillance of roommates in nosocomial cases, which we believe have played a key role in the results and need to be confirmed in future research. In addition, encouraging results such as the decrease in mortality in nosocomial cases, without statistical significance in our study, could be confirmed with a larger sample size in the coming seasons.

We can attribute much of the reduction in nosocomial transmission to prevention and control measures incorporated in our surveillance system since vaccination coverage of health professionals decreased slightly between seasons, vaccination levels of patients before and after the intervention were comparable and virus strains did not differ markedly. For two fundamental reasons, we believe that these improvements could be implemented in other acute care hospitals. First, the proposed measures are easy to implement and do not require additional economic, structural, or human resources. Moreover, the intervention was implemented in an environment of demand and usual care pressure during the study period.^34^, ^35^ However, the intervention does require the determined commitment and coordination of the management team of the center and the different professional groups involved in the process. A relatively simple strategy can have a great impact on patient safety.

The economic impact of our results is undeniable. For every 100 patients attended when the real‐time surveillance system was implemented, more than 7 cases of nosocomial influenza transmission were averted. Previous experience in similar care complexes placed the average cost for hospitalized patients at 6236 euros.^7^ To this, we must add the indirect costs, since each case of influenza is associated with 5‐6 days of limited activity and about 3 days of work absenteeism.^36^ Cost analysis is required to determine the specific economic impact of real‐time surveillance systems such as the one we propose.

Both the chosen design and the subject under study entail a series of limitations. Developing a before‐and‐after study in a single center for two seasons does not allow us to know whether the effectiveness of the intervention is maintained over time and makes it difficult to generalize the results. In addition, multiple factors can influence mortality and nosocomial transmission of influenza viruses, such as their pathogenicity, vaccine effectiveness or global and weekly incidence rates, that complicate the determination of the specific effect of a preventive measure. Despite knowing the proportion of patients who are isolated after a positive PCR in the emergency department, we do not have more data to assess the general adherence of professionals to the new surveillance system. Finally, focusing on patient‐to‐patient transmission has prevented us from analyzing other possible sources of infection, such as visitors and healthcare workers.

The results obtained reveal the impact of nosocomial transmission of influenza in a tertiary hospital, pointing out the need to continue actively promoting the vaccination of health professionals, but not forgetting that other prevention and control measures, integrated in surveillance systems, can contribute significantly to reducing virus transmission.

## CONFLICT OF INTEREST

The authors declare no conflicts of interest.
