# Supplementary figures and images for: Real‐time surveillance systems: Applicability for the control of influenza in acute care
Source: Influenza Other Respir Viruses. 2020 Mar 2;14(3):331–9. doi: 10.1111/irv.12720 (PMC7182607; doi:10.1111/irv.12720)

**APPENDIX I**. An example of a translated and anonymized daily report


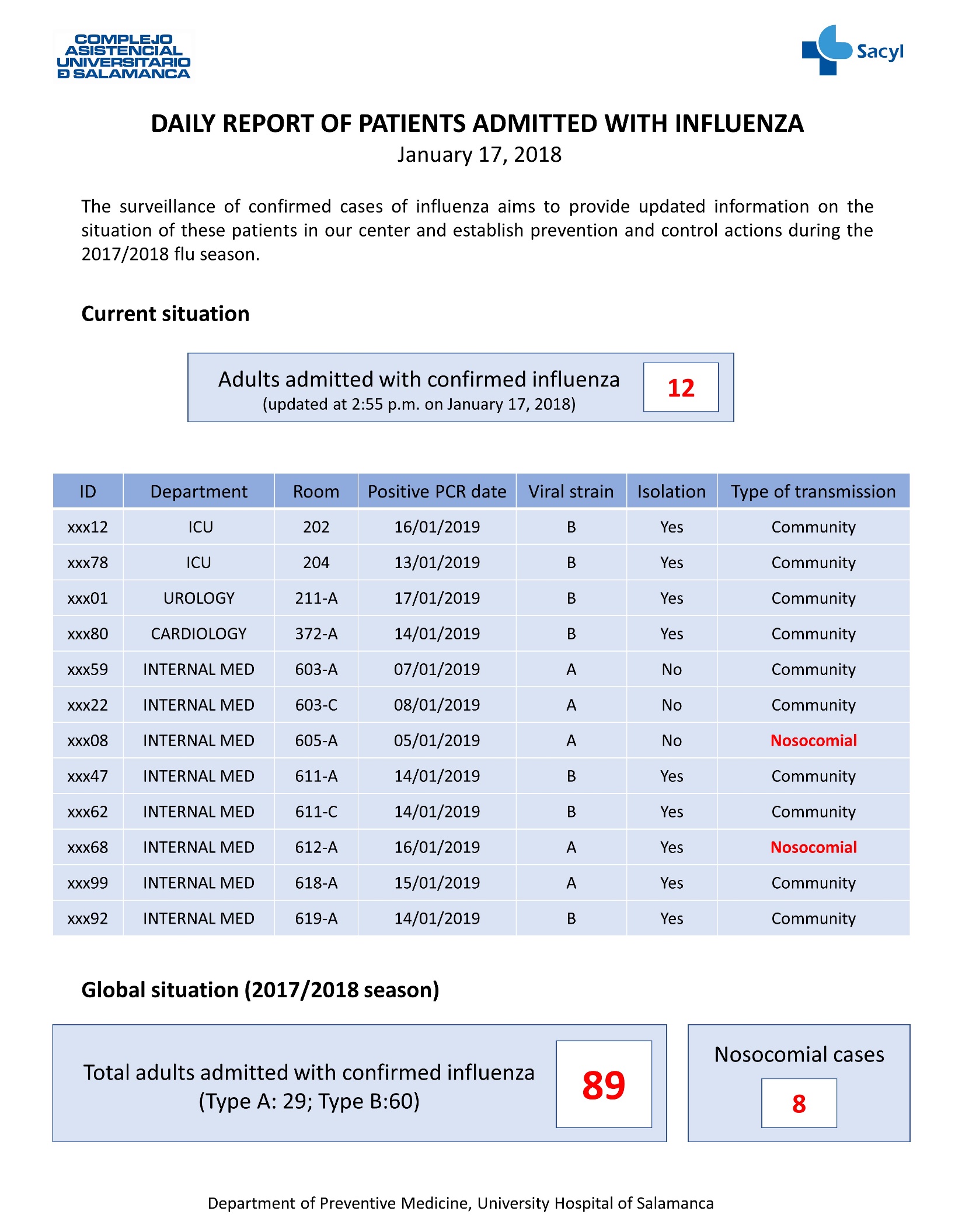

Supplement: Supplementary file 1 [file IRV-14-331-s001.docx]
